# Stream-based Hebbian eigenfilter for real-time neuronal spike discrimination

**DOI:** 10.1186/1475-925X-11-18

**Published:** 2012-04-10

**Authors:** Bo Yu, Terrence Mak, Xiangyu Li, Leslie Smith, Yihe Sun, Chi-Sang Poon

**Affiliations:** 1 Tsinghua National Laboratory for Information Science and Technology, Institute of Microelectronics, Tsinghua University, Beijing 100084, China; 2 School of Electrical, Electronic and Computer Engineering, Newcastle University, Newcastle upon Tyne NE47RU, UK; 3 Institute of Neuroscience, Newcastle Biomedicine, Newcastle University, Newcastle upon Tyne NE17RU, UK; 4 Department of Computing Science and Mathematics University of Stirling, Stirling FK9 4LA, UK; 5 Harvard-MIT Division of Health Sciences and Technology, MIT, Cambridge MA 02139, USA

**Keywords:** Brain-machine interface, Spike sorting, Hebbian eigenfilter, Hardware, Principal component analysis

## Abstract

**Background:**

Principal component analysis (PCA) has been widely employed for automatic neuronal spike sorting. Calculating principal components (PCs) is computationally expensive, and requires complex numerical operations and large memory resources. Substantial hardware resources are therefore needed for hardware implementations of PCA. General Hebbian algorithm (GHA) has been proposed for calculating PCs of neuronal spikes in our previous work, which eliminates the needs of computationally expensive covariance analysis and eigenvalue decomposition in conventional PCA algorithms. However, large memory resources are still inherently required for storing a large volume of aligned spikes for training PCs. The large size memory will consume large hardware resources and contribute significant power dissipation, which make GHA difficult to be implemented in portable or implantable multi-channel recording micro-systems.

**Method:**

In this paper, we present a new algorithm for PCA-based spike sorting based on GHA, namely stream-based Hebbian eigenfilter, which eliminates the inherent memory requirements of GHA while keeping the accuracy of spike sorting by utilizing the pseudo-stationarity of neuronal spikes. Because of the reduction of large hardware storage requirements, the proposed algorithm can lead to ultra-low hardware resources and power consumption of hardware implementations, which is critical for the future multi-channel micro-systems. Both clinical and synthetic neural recording data sets were employed for evaluating the accuracy of the stream-based Hebbian eigenfilter. The performance of spike sorting using stream-based eigenfilter and the computational complexity of the eigenfilter were rigorously evaluated and compared with conventional PCA algorithms. Field programmable logic arrays (FPGAs) were employed to implement the proposed algorithm, evaluate the hardware implementations and demonstrate the reduction in both power consumption and hardware memories achieved by the streaming computing

**Results and discussion:**

Results demonstrate that the stream-based eigenfilter can achieve the same accuracy and is 10 times more computationally efficient when compared with conventional PCA algorithms. Hardware evaluations show that 90.3% logic resources, 95.1% power consumption and 86.8% computing latency can be reduced by the stream-based eigenfilter when compared with PCA hardware. By utilizing the streaming method, 92% memory resources and 67% power consumption can be saved when compared with the direct implementation of GHA.

**Conclusion:**

Stream-based Hebbian eigenfilter presents a novel approach to enable real-time spike sorting with reduced computational complexity and hardware costs. This new design can be further utilized for multi-channel neuro-physiological experiments or chronic implants.

## Background

Recently, multi-electrode arrays (MEAs) have become increasingly popular for neuro-physiological experiments *in vivo *[1-4] or *in vitro *[5-8]. Compared with other methods of signal acquisition, such as Electroencephalography (EEG) [9] and Electrocorticographical (ECoG) [10], MEAs provide the capability of recording neuronal spikes from specific regions of the brain with high signal-to-noise ratio [1,5]. The substantial temporal and spatial resolutions provided by MEAs facilitate the studies of neural network dynamic [11], plasticity [12], learning and information processing [13] and the developments of high performance brain-machine interface (BMI) for emerging applications, such as motor rehabilitation for paralyzed or stroke patients [14-16].

Neuronal spike trains recorded by electrodes encompass noises introduced by measurement instruments, interferences from other vicinity neurons and action potentials from unknown number of neurons. Neural signal processing that extracts useful information from noisy spike trains is necessary for spike information decoding and neural network analysis in subsequent processes. In most MEA based systems, especially the MEA based brain-machine interface (BMI), spike sorting that discriminates neuronal spikes to corresponding neurons is among the very first steps of signal processing [17-19] and its correctness significantly affects the reliability of the subsequent analysis [20].

### PCA-based spike sorting

**Figure 1:**
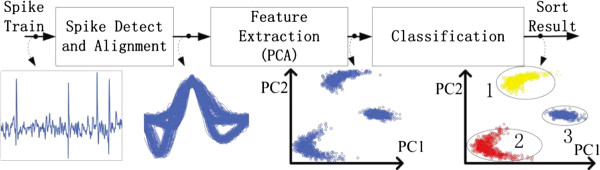
**The procedure of PCA-based spike sorting**.

Principal component analysis (PCA) is an effective and automatic spike sorting method. PCA-based spike sorting calculates principal components (PCs) of a group of neuronal spikes, and uses the first 2 or 3 PCs to constitute a feature space. Most variance of spikes can be captured by the feature space. When projecting neuronal spikes into the feature space, inherent characters of neuronal spikes are highlighted, and several clusters composed by different neuronal spikes can be observed and differentiated. A complete PCA-based spike sorting flow, including spike detection, peak alignment, PCA-based feature extraction, and clustering is illustrated by Figure 1. Firstly, spike detection [21,22] distinguishes neuronal spikes from background noises according to a pre-trained threshold. Detected high dimensional spikes are then aligned at their peak point for the PCA. PCs of aligned neuronal spikes that are orthogonal to each other can be obtained through PCA-based feature extraction. The first two or three PCs are used to constitute a low dimensional feature space. Aligned spikes are projected into the feature space by the dot production between spikes and each PC. In the feature space, dots represent aligned neuronal spikes and each cluster represents a prospective neuron. At last, clustering algorithms are employed to differentiate clusters in the feature space and assign dots (spikes) to their closest cluster (neuron).

Calculating PCs requires computationally expensive operations, including covariance matrix calculation and eigenvalue decomposition. Besides, a large volume of neuronal spikes needs to be temperately stored for training PCs. Large size memories are therefore required. While large computational complexity and memory usage are not issues for recording and analysis of 100 channel systems [23], they are critical concerns for systems with large number of channels (> 1000), and especially for implantable systems, which have tight constraints of hardware size and power consumptions.

### GHA for calculating PCs of neuronal spikes

In our previous work [24], general Hebbian algorithm (GHA), so-called Hebbian eigenfilter, which presents an efficient approach for realizing PCA, was proposed for calculating the leading PCs of neuronal spikes. Let $\vec{\text{x}}\text{(i)}$, i ∈ [1, n] be *n *aligned spikes. Each aligned spike is *d *dimension (containing *d *sample points), *i.e. *$\vec{\text{x}}\text{(i)}={\left[{\text{x}}_{\text{1}}\text{(i),}{\text{x}}_{\text{2}}\left(\text{i}\right),...,{\text{x}}_{d}\left(\text{i}\right)\right]}^{T}$. Let *l *be the dimension of feature space (the number of extracted principal components), *η *be the learning rate, $\vec{W}\left(\text{j}\right)\text{ = }{\left[{\vec{W}}_{1}\left(\text{j}\right),{\vec{W}}_{2}\left(\text{j}\right),...,{\vec{W}}_{d}\left(\text{j}\right)\right]}^{T}$ be a *l × d *synaptic weight matrix that is initialized to $\vec{W}\left(1\right)$, and j be the iteration index. GHA for calculating the first *l *principal components of *n *aligned neuronal spikes is summarized in Table 1. Step 1 initializes synaptic weights and the learning rate. The mean vector of *n *aligned spikes, $\vec{\mu }$, is calculated in Step 2. After the mean vector is available, the mean vector is subtracted from all aligned spikes in Step 3. After that, iteration learning will be performed on zero-mean spikes. In the iteration learning, $\text{LT}\left[\vec{\text{m}}\right]$ is an operator that sets all the elements above the diagonal of matrix, $\vec{\text{m}}$, to zeros. The algorithm stops when the iteration step, *j*, equals to *N*. If *N *is large enough and the learning rate is appropriate [25], $\vec{\text{W}}\text{(j)}$ will converge to the *l *most significant principal components of input spikes.

**Table 1:** GHA-basedspike feature extraction

Input:
Neuronal spikes, $\vec{\text{x}}\text{(i)}$;

Initial synaptic weight, $\vec{\text{W}}\text{(1)}$;

Learning rate, *η *;

**Output:**

Principal components of neuronal spikes, $\vec{\text{W}}\text{(N)}$;

*1*. *Initialize synaptic weight *$\vec{\text{W}}\text{(1)}$*and learning rate η*, *j = *1

*2*. *Calculate the mean vector of the aligned spikes*

$\vec{\mu }=\sum_{\text{i}=1}^{n}\vec{\text{x}}\text{(i)/n}$

*3*. *Zero-mean transformation*

$\vec{\text{x}}\text{(i)}=\vec{\text{x}}\left(\text{i}\right)-\vec{\mu }\;1\leq \text{i}\leq \text{n}$

*4*. *Perform Hebbian learning on zero-mean data*

$\vec{\text{y}}\text{(j)}=\vec{\text{W}}\text{(j)}\vec{\text{x}}\text{(i)}$

$\text{LT(j)}=\text{LT}\left[\vec{\text{y}}\text{(j)}{\vec{\text{y}}}^{\text{T}}\text{(j)}\right]$

$\text{dW(j)}=\eta \left(\vec{\text{y}}\text{(j)}{\vec{\text{x}}}^{\text{T}}\left(\text{i}\right)-\text{LT(j)}\vec{\text{W}}\text{(j)}\right)$

$\vec{\text{W}}\text{(j + 1)}=\vec{\text{W}}\text{(j) + dW(j)}$

*5*. *If j= N, the algorithm stops, otherwise j = j+ *1*, go to step 4*

Compared with conventional PCA algorithms, GHA does not involve complicated matrix computations, such as covariance matrix calculation and eigenvalue decomposition. Also, the algorithm has the ability to filter a specified number of most significant PCs. Since most variances of aligned spikes are captured by the first few PCs, computing the leading PCs can effectively reduce a lot of computational efforts.

Although GHA eliminates computationally expensive operations, large memory resources are still required in the training procedure. Large size hardware memories consume significant hardware resources and power, which makes GHA impractical for systems with tight constraints on hardware resources and power consumption.

In this paper, a new algorithm, namely stream-based Hebbian eigenfilter, is proposed to mitigate the problem associated with the large memory required in the training procedure. Neural signals can be regarded as pseudo-stationary in considering of a short recording period and relatively stable recording condition. The stream-based algorithm exploits the pseudo-stationary characteristic of neural spikes and, thus eliminates the need for temporary storage in the Hebbian learning. In order to justify the streaming method and evaluate the performance of the stream-based eigenfilter, both clinical and synthetic spike trains were used in this study. The accuracy of spike sorting using the stream-based Hebbian eigenfilter was evaluated by comparing with conventional PCA algorithms. The improvements in memory size and power consumption were rigorously evaluated using FPGA devices. The hardware performance of our method was also compared with FPGA-based PCA. Evaluation results show that our proposed approach mitigates the expensive hardware requirement of PCA, and further enables real-time multi-channel recording systems and future BMI systems.

## Method

### Stream-based Hebbian eigenfilter

As shown in the definition of GHA-based feature extraction (Table 1), Hebbian learning is defined to perform on a group of spikes of which mean is zero. Since zero-mean transformation cannot start until the mean is available, memories are required to temporarily store all the aligned spikes when computing the mean vector. Therefore, the same set of neuronal spikes can be used in the subsequent zero-mean transformation and Hebbian learning. The memory size needed for buffering aligned spikes can be formulated by Eq. 1, where *n *is the number of spikes for learning, *d *is the number of sample points of each spike, and *w *is the hardware word length of each sample point. For a quantitative estimation of the memory requirement, we chose values of these parameters based on commonly used recording and training conditions [19,26]. 25 KHz of sampling rate and 64 sampling points per spikes (each spike spans around 2.5 ms) were used for our estimation. In general, thousands neuronal spikes are used for training PCs [19]. Suppose that the number of neuronal spikes for training is 1024, and each sample is 16 bits, 1 million bits memories are required for storing spikes. Note that this is only for one channel training. Even larger memories are required for the multi-channel scheme.

(1) $$\text{Me}{\text{m}}_{\text{spikes}}=\text{n}\times \text{d}\times \text{w}$$

Memory resources are expensive in terms of hardware design, especially for implantable or portable applications. Large size memory will consume large portions of hardware resources in hardware constrained systems. Reducing storage resources allows integrating more computing resources in a single device, and further enables parallel processing for a large number of channels at the same time. In addition, large memories contribute significant power consumption and thermal dissipation of hardware, which should be minimized for implantable or portable applications. In this section, we proposed a method, so-called stream-based Hebbian eigenfilter, to reduce the extremely large memory associated with Hebbain training by utilizing the pseudo-stationary property of neuronal spikes.

Recorded neural signals have non-stationary nature which is mainly due to the relative movements between the recording electrodes and the recorded neurons. Also, the magnitude of spike could change during excitation block or post inhibitory rebound excitation or spike frequency adaptation, and the shape of the spike could change when other ion channels are recruited, such as calcium-dependent potassium channels. However, neural signals can be regarded as pseudo-stationary in considering of a short recording period and relatively stable condition, simply because the chance of disturbance is small.

During a period of time, in which pseudo-stationarity exists, the shape of a neuronal spike stays relatively fixed and can only be disturbed by various noises. This similarity in recorded neuronal signals can be utilized to reduce the temporary storage requirement. That the same operation performed on signals from different recording periods can lead to similar results due to the similarity of signals. For mean calculation, it means that mean values of spikes from different recording periods are similar under the pseudo-stationary property. Hebbian eigenfilter can use the mean vector obtained from the previous recording period as an approximate mean of current spikes when performing zero-mean transformation and Hebbian learning. Therefore, there is no need to store a certain subset of data for both mean calculation and zero-mean transformation, and the corresponding memory requirement is eliminated.

The algorithm of stream-based Hebbian eigenfilter for spike sorting is presented in Table 2. It has the same initialization scheme with the non-streaming algorithm, and then calculates the mean vector of *n *neuronal spikes (step 2 in Table 2). Different from the non-streaming algorithm for spike sorting, the spikes for mean calculation are discarded after use. After mean calculation, zero-mean transformation and Hebbian learning are performed on the obtained mean vector and the subsequent spikes (step 3 and 4 in Table 2). Since mean calculation and zero-mean transformation are performed on different data sets, there is no explicit storing requirement associated with mean operations and Hebbian learning in the stream-based algorithm.

**Table 2:** Algorithm of stream-based Hebbian Eigenfilter for spike sorting

Input:
Neuronal spikes, $\vec{\text{x}}\text{(i)}$;

Initial synaptic weight, $\vec{\text{W}}\text{(1)}$;

Learning rate, *η*;

**Output:**

Principal components of neuronal spikes, $\vec{\text{W}}\text{(N)}$;

*1*. *Initialize synaptic weight *$\vec{\text{W}}\text{(1)}$*and learning rate η, j = 1*

*2*. *Calculate the mean vector of n aligned spikes*

*a. i= 1*, $\vec{\mu }=0$

*b. When new spike *$\vec{\text{x}}\text{(i)}$*arrives *$\vec{\mu }=\vec{\mu }+\vec{\text{x}}\text{(i)}$

*c. If i equals to n, $\vec{\mu }=\vec{\mu }/n$, and go to step 3. Otherwise i = i + 1, go to Step 2.b*

*3*. *When new spike arrives, subtract the mean vector obtained from step 2*

$\vec{\text{x}}\text{(i)}=\vec{\text{x}}\text{(i)}-\vec{\mu }\;\text{i}>\text{n}$

*4*. *Perform Hebbian *x *learning on *$\vec{\text{x}}\text{(i)}$

$\vec{\text{y}}\text{(j)}=\vec{\text{W}}\text{(j)}\vec{\text{x}}\text{(i)}$

$\text{LT(j)}=\text{LT}\left[\vec{\text{y}}\text{(j)}{\vec{\text{y}}}^{\text{T}}\text{(j)}\right]$

$\text{dW(j) = }\eta \left(\vec{\text{y}}\text{(j)}{\vec{\text{x}}}^{\text{T}}\left(i\right)-\text{LT(j)}\vec{\text{W}}\text{(j)}\right)$

$\vec{\text{W}}\text{(j + 1)}=\vec{\text{W}}\text{(j) + dW(j)}$

*5*. *If j= N, the algorithm stops, otherwise j = j + *1*, go to step 3*

### Evaluation method

Stream-based Hebbain eigenfilter uses data sets from different recording periods for mean calculation and Hebbian learning. It is an approximate method based on the precondition of pseudo-stationarity of neuronal spikes. In order to justify the pseudo-stationarity, we evaluated the discrepancy in mean and PCs obtained by stream-based eigenfilter and conventional PCA. In order to test the performance of the eigenfilter in the spike sorting scenario, the accuracy of spike sorting using stream-based Hebbain eigenfilter was further evaluated, and compared with that using conventional PCA algorithm. In addition, both streaming and non-streaming eigenfilter were implemented on FPGAs to evaluate the memory and power consumption reduced by utilizing the stream-based method.

#### Testing data

In order to verify the streaming method, realistic neuronal spike shapes, background noises and vicinity neuronal interference should be taken into account in the evaluation. Both clinical data [27] and sophisticated synthetic spikes [27] were utilized for the evaluation. These synthetic spike trains accurately model various background noises and neuronal spikes profile that appear at single-channel clinical recordings.

For quantitatively evaluating the performance of the Hebbian spike sorting algorithm, spike trains with known spike times and classifications should be employed. Clinical extracellular recording with realistic spike shapes and noise interferences is one option for qualitative studies. However, it is difficult to determine the precise classifications and the source of the spike from clinical measurement and these are the "ground truth" for any effective quantitative evaluation. Although these recordings can be further manually annotated by experts, it has been shown that manually clustered spikes have relative low accuracy and reliability [28]. For these reasons, synthetic spike trains were utilized to quantify the performance of the proposed algorithm.

**Figure 2:**
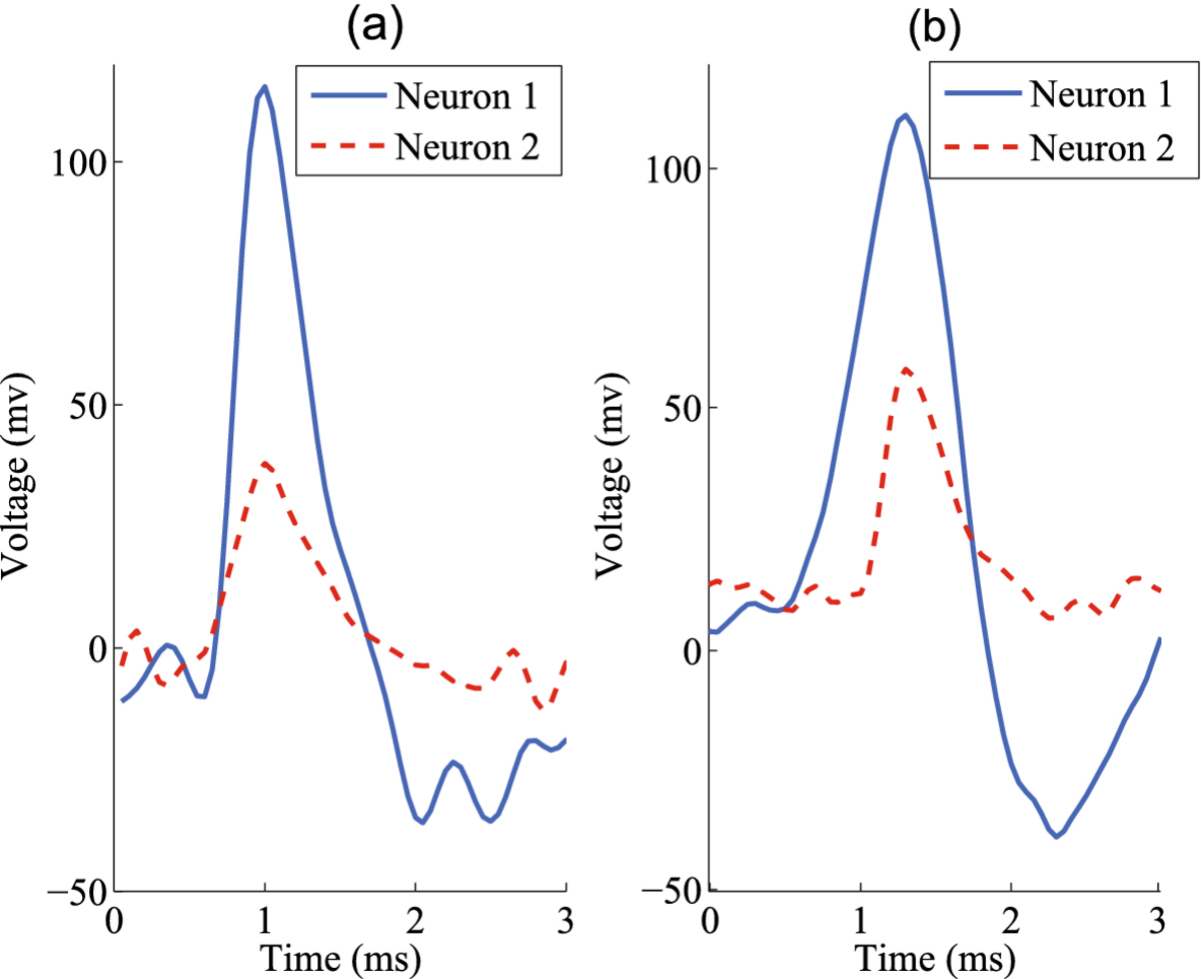
**(a) Clinical and (b)synthetic spike waveform of two neurons **[24].

Both baseline [29] and sophisticated synthetic spike trains [27] were employed for the quantitative evaluation. These benchmarks were used to maximize representative different scenarios in real experiments. The baseline spike trains were generated from spike synthesis tool [29]. The tool accurately models factors affecting extracellular recordings, such as ion channels of the membrane, the electrical resistance and capacitance of the membrane, the extended spiking neural surface, back ground noises and inference from other neurons, and provides an approximation of realistic clinical data. Figure 2 shows both the clinical and synthetic spike shapes. More importantly, parameters, such as the number of neurons contributing to the spike train, the waveform of neuronal spike, signal to noise ratio and the firing rate, can all be specified in the tool. Through adjusting these parameters, various spike trains can be generated for quantitative evaluations. For our evaluation, three groups of spike trains containing two, three and four neurons were generated. White noise and artifacts noises contributed by background neurons were considered when generating noisy synthetic spike trains. Each group contains spike trains with 11 different noise levels. The level of noises is quantified by the SNR (signal-to-noise ratio). Under the same noise level, a group of spike trains with neuron's firing rate from 5 Hz to 50 Hz was generated. All the data sets are 100 s in length and generated at a sampling rate of 25 KHz. These spike trains provide ideal testing benchmarks to evaluate the proposed algorithm with a variety of noise immunity and realistic background noise. Because our method differentiates neuronal spikes according to spike profiles, it is not effective for bursting spikes that appear as concatenated and with decreasing amplitude. Although bursting spikes can be identified and ruled out with the help of inter-spike-interval histograms and cross-correlograms, addressing how to combine these methods with our work is beyond the scope of the paper. In this paper, we do not take bursting spikes into account.

#### Accuracy evaluation

As shown in Table 2 stream-based algorithm uses the mean vector of one data set to estimate the mean vector of the following spikes (belonging to another data set). This approximation of mean calculation is based on pseudo-stationary property of neuronal spikes. Let *d *be the dimensionality of the mean vector, ${\vec{\mu }}_{\text{j}}$ be the mean vector of data set *j*, and *μ*_j,i _represents the *i*-th elements of the mean vector of data set *j*. We used the difference (error) between mean vectors obtained from two different data sets to justify the pseudo-stationarity and the approximation. The difference (error) of mean is defined by Eq. 2. The value of the difference (error) indicates the accuracy of the streaming method for mean estimation. The smaller the error is, the more accurate the estimation is. When the difference is zero, ${\vec{\mu }}_{2}$ can be accurately estimated from ${\vec{\mu }}_{1}$.

(2) $$\text{Erro}{\text{r}}_{\text{mean}}=\frac{\sum_{\text{i}=1}^{\text{d}}|\mu_{1,\text{i}}-\mu_{2,\text{i}}|}{\sum_{\text{i}=1}^{\text{d}}|\mu_{2,\text{i}}|}$$

Further, we evaluated the deviation between PCs obtained by the stream-based Hebbian eigenfilter and conventional PCA. For PCA-based spike sorting, the direction of PCs determines the feature space that is critical to the accuracy of classification. We therefore used the angle between synaptic weights and PCs to define the deviation (error) between PCs obtained by eigenfilter and PCA, as shown in Eq. 3. In the equation, $\vec{\text{W}}$ and $\text{P}\vec{\text{C}}$ are synaptic weights and PCs obtained by Hebbian and conventional PCA methods respectively. When the two vectors have the same direction or the opposite direction, the result will equal to zero. Otherwise a value in [0,1) is obtained.

(3) $$\text{Erro}{\text{r}}_{\text{PC}}=1-\left|\vec{\text{W}}\text{.PC}\right|$$

Finally the accuracy of spike sorting using stream-based Hebbian eigenfilter was evaluated in our work. Besides Hebbian eigenfilter, NEO based detection algorithm [22] and K-means clustering algorithm [30] were employed in our evaluation to complete spike sorting algorithms. We used the true positive rate (TPR) and the false positive rate (FPR) to evaluate the performance of our algorithm. The true positive rate is defined by Eq. 4, where *K *is the number of estimated clusters, and *Num_correct_classified_spikes,i _*and *Num_spikes,i _*stand for the number of correctly classified spikes of neuron *i *and the total number of spikes of neuron *i*, respectively.

(4) $$\text{TPR = }\frac{1}{\text{K}}\overset{\text{K}}{\underset{\text{i}=1}{\sum }}\frac{\text{Nu}{\text{m}}_{\text{correct\_classified\_spikes;i}}}{\text{Nu}{\text{m}}_{\text{spikes;i}}}$$

The false positive rate is defined by Eq. 5, where *Num_false_classified_spikes,i _*and *Num_false_spikes,i _*stand for the number of false spikes (not belonging to neuron *i*) assigned to neuron *i *and the total number of false spikes for neuron *i*, respectively.

(5) $$\text{FPR = }\frac{1}{\text{K}}\overset{\text{K}}{\underset{\text{i}=1}{\sum }}\frac{\text{Nu}{\text{m}}_{\text{false\_classified\_spikes;i}}}{\text{Nu}{\text{m}}_{\text{false\_spikes;i}}}$$

#### Computational complexity evaluation

In order to quantitatively evaluate the computational complexity, we used the number of operations and hardware memories required by an algorithm in the study. By comparing stream-based Hebbian eigenfilter with GHA and a group of traditional PCA algorithms, the computationally economical property of the proposed method was demonstrated. The operation and memory counts of stream-based Hebbian eigenfilter were obtained according to the algorithm shown in Table 2. The computational complexity of GHA was derived according to Table 1. Conventional PCA algorithms are composed by covariance matrix calculation and eigenvalue decomposition of covariance matrix. For eigenvalue decomposition, three commonly used symmetric eigenvalue decomposition methods were taken into account for our comparison, which were the orthogonal iteration [31], the symmetric QR algorithm [31] and the Jacobi method [31]. Operation and memory counts for covariance matrix calculation and symmetric matrix decomposition algorithms were derived according to standard texts on matrix computation [31].

### Algorithm implementation

The algorithm of stream-based eigenfilter was developed under Matlab R2009b. Matlab built-in functions, *princomp*, was used as the conventional PCA routine. For evaluating the performance of spike sorting using stream-based Hebbian eigenfilter and PCA, NEO-based spike detection algorithm was developed using Matlab, and Matlab built-in functions, *kmeans*, was used for K-means clustering.

### Hardware implementation

Xilinx FPGA was used for hardware implementation and evaluation. The targeting device is Xilinx Spartan6 FPGA (xc6slx75t). Xilinx System Generator was our hardware design tool, which is a schematic based design tool. Working in Matlab Simulink environment makes System Generator easy for hardware-software co-design and hardware verification. We obtained the hardware resources usage through Xilinx ISE tool. The hardware power was obtained from Xilinx Xpower tool.

**Figure 3:**
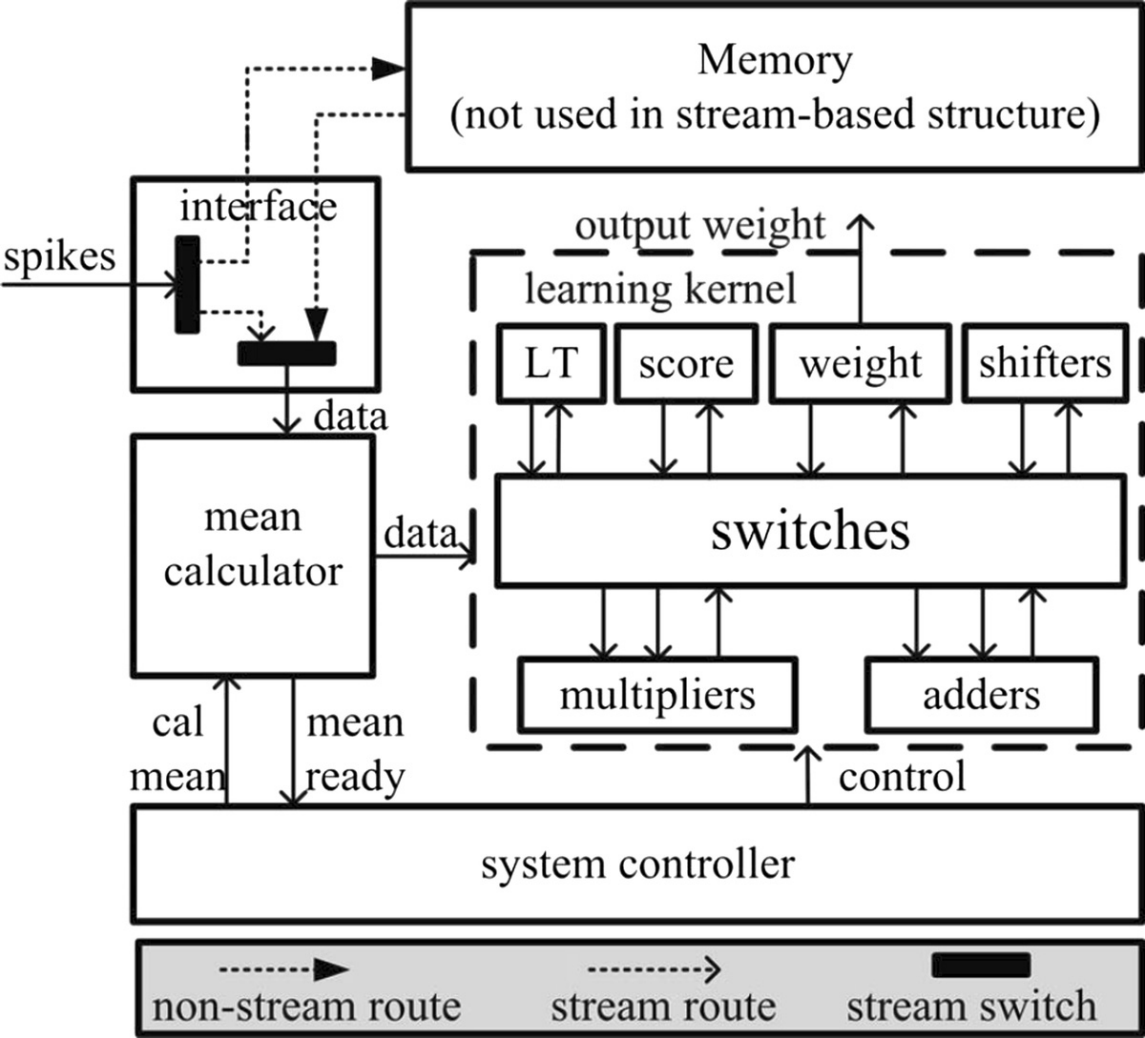
**The hardware architecture of Hebbian eigenfilter**.

Both streaming and non-streaming Hebbian eigenfilter were implemented on the FPGA. Figure 3 shows the architecture of hardware Hebbian eigenfilter. It is an example that the first three principal components are filtered. It consists "learning kernel", "system controller", "mean calculator", "interface" and "memory". Memory block only exists in the non-streaming structure. "System controller" controls the operation of the whole system. "Learning kernel" performs learning operations and consists of arithmetic units, storing units and switchers. "LT" stores the result of $\text{LT}=\text{LT}\left[\vec{\text{y}}{\vec{\text{y}}}^{\text{T}}\right]$, "score" stores result of $\vec{\text{y}}=\vec{\text{W}}\vec{\text{x}}$, "weight" stores the three synaptic weights. These synaptic weights are updated concurrently. Shifters are used when multiplying a small learning rate has to be performed. Switchers route the signals between arithmetic unit and storage elements. "Mean calculator" calculates mean of data before mean is ready. After mean is ready, "mean calculator" subtracts mean from data and sends mean centered data to "learning kernal". In the non-streaming Hebbian eigenfilter, "interface" write the input streaming data to the memory and read data from memory for mean calculation and Hebbian learning. In the stream-based Hebbian eigenfilter, "interface" directly forwards the input data to "mean calculator".

**Figure 4:**
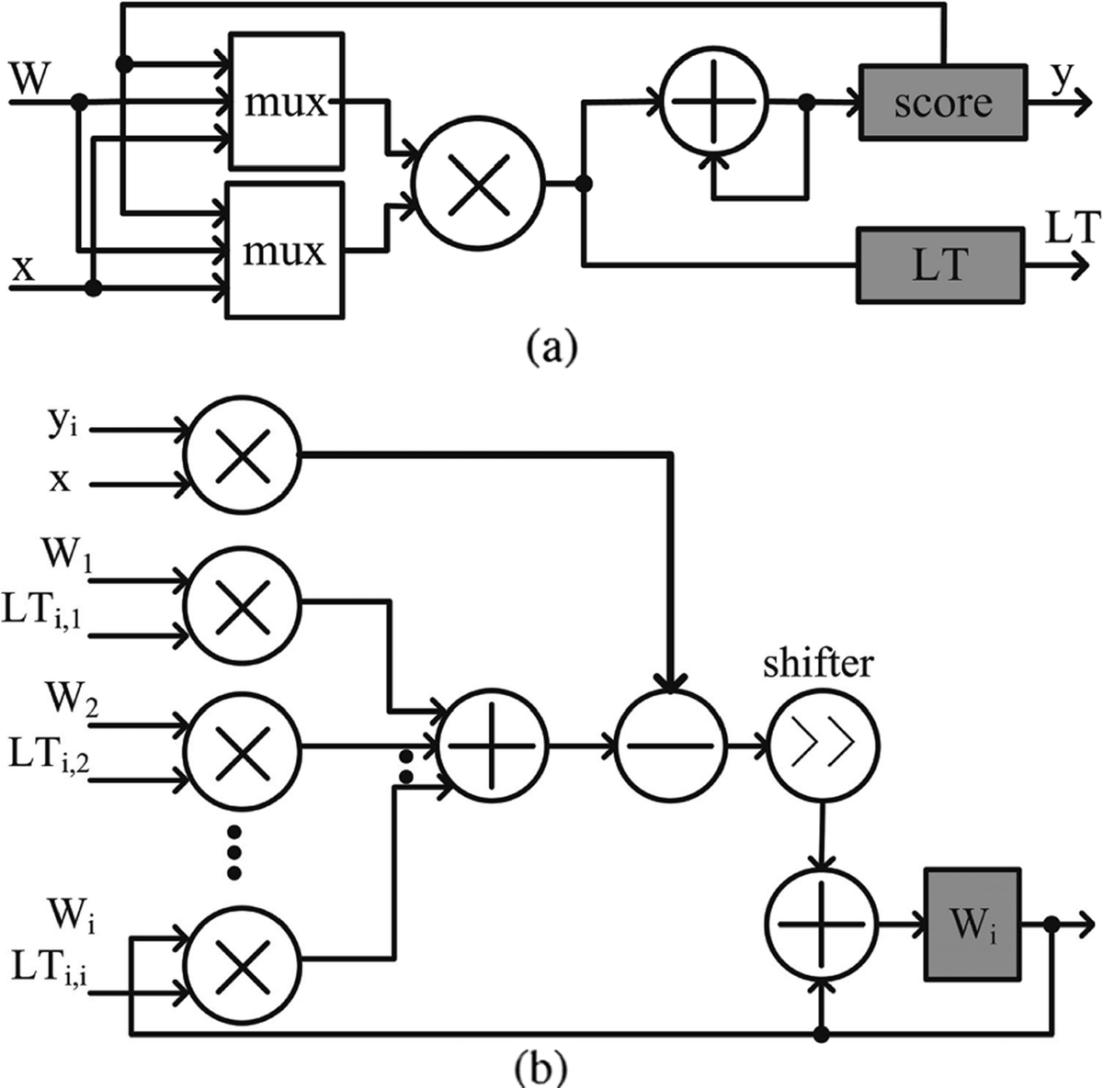
**(a) The data path for calculating score $\left(\vec{\text{y}}=\vec{\text{W}}\vec{\text{x}}\right)$ and the lower triangular matrix $\vec{\text{y}}{\vec{\text{y}}}^{\text{T}}$**. (b) The data path for updating synaptic weights, ${\vec{\text{W}}}_{\text{i}}$.

The data path for computing the score, $\vec{\text{y}}$, and $\text{LT}=\text{LT}\left[\vec{\text{y}}{\vec{\text{y}}}^{\text{T}}\right]$ is illustrated by Figure 4(a). The multipliers and accumulators (adders) implement the dot product between input spikes and synaptic weights. The results of dot products are stored in the "score". After the scores are obtained, multipliers are re-used for calculating the lower triangular matrix of $\vec{\text{y}}{\vec{\text{y}}}^{\text{T}}$. The results are stored in the "LT". Figure 4(b) shows the data path for updating the *i*-th synaptic weight, ${\vec{\text{W}}}_{\text{i}}$. Updated results are stored in registers "W_i_".

## Results and discussion

### Accuracy evaluation

**Figure 5:**
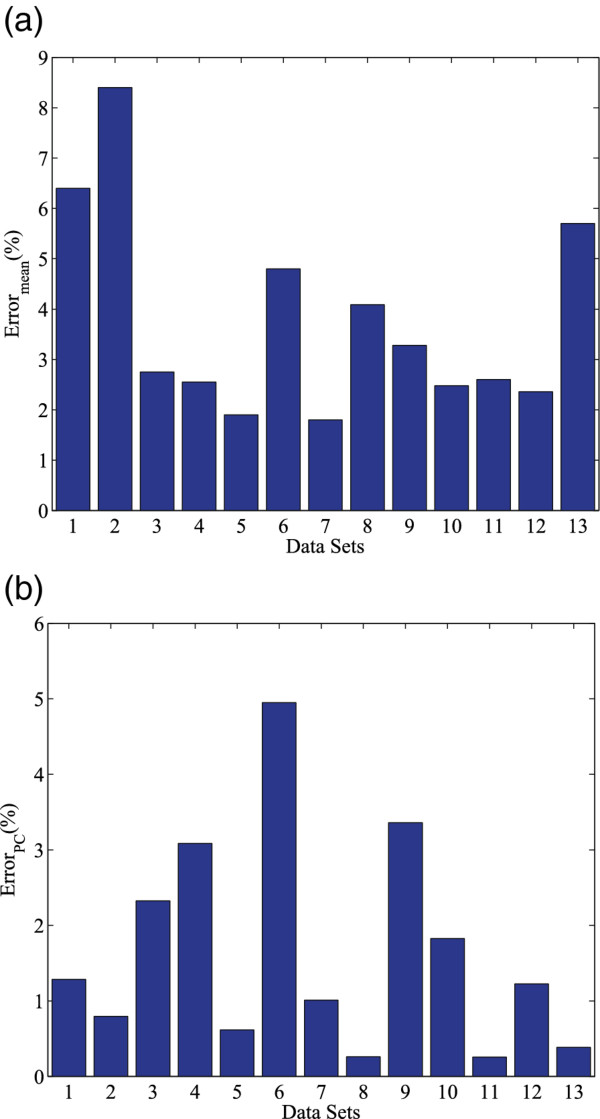
**Error evaluation of steam-based Hebbian eigenfilter when compared to PCA**. **(a) **Errors for mean calculation and, **(b) **principal components calculation. Benchmark #1 ~ #12 are obtained from [27], #13 is clinical data from [27].

In general, thousands of spikes are used in PC training procedure [19]. In our evaluation, we suppose 1024 spikes are used for training. For stream-based method, we used the first 1024 spikes (data set 1) for mean calculation and the following 1024 spikes (data set 2) for Hebbian learning. Figure 5(a) and 5(b) show the evaluation results of differences (errors) between mean vectors of the two data sets (defined by Eq. 2) and deviations of PCs obtained by stream-based eigenfilter and PCA (defined by Eq. 3). Clinical data and synthetic spikes from [27] were used in the evaluation. The average error of mean vector is 3.7%. The average deviation of the first two PCs is 1.7%. From the results we can see that the error of mean vector introduced by the streaming estimation is small. This small error of mean vector leads to small discrepancy in PCs.

**Figure 6:**
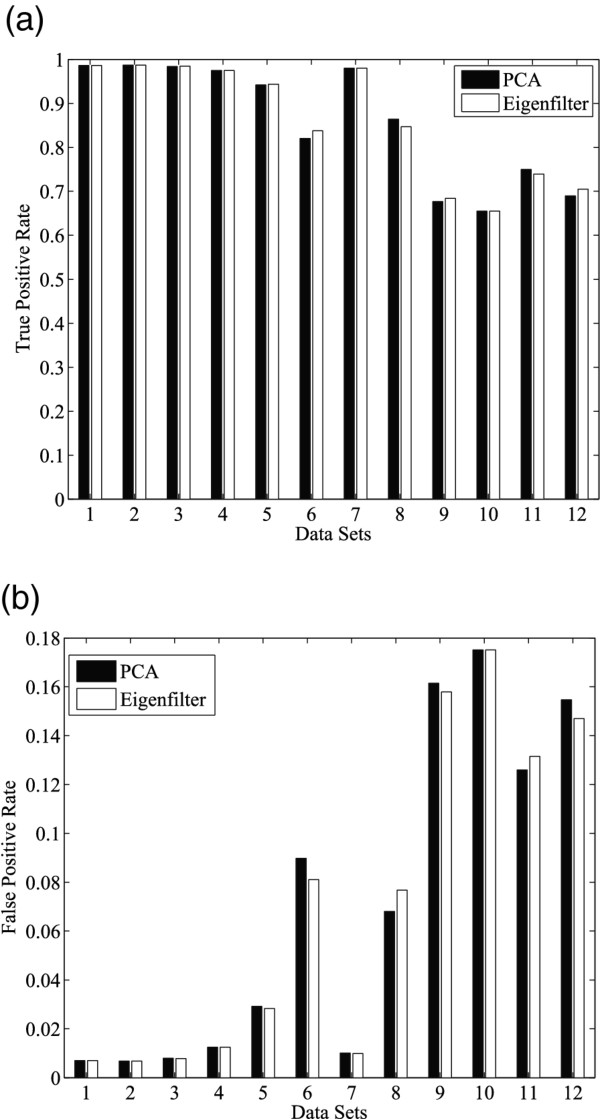
**(a) True positive rate and (b) false positive rate of spike sorting using PCA (Matlab princomp) and stream-based Hebbian eigenfilter**. Benchmarks #1 ~ #12 are obtained from[27].

We further evaluated the performance of Hebbian-based spike sorting algorithm and compared with that using conventional PCA. For stream-based Hebbian eigenfilter, we let N equal to 1024, which is the number of spikes used for mean calculation and Hebbian learning. The initializing learning rate equals to 0.1 and the weights equal to 0.5. It has been shown in the experiments that the convergence of the eigenfilter is relatively independent to the initial synaptic weights. However, the learning rates would contribute to the speed of the convergence, as reported in [24]. Both true positive rate and false positive rate of classification were considered in our evaluation, as shown in Figure 6(a) and 6(b). The results show that stream-based Hebbian eigenfilter can achieve nearly the same performance as PCA-based sorting in terms of the accuracy of spike classification.

**Figure 7:**
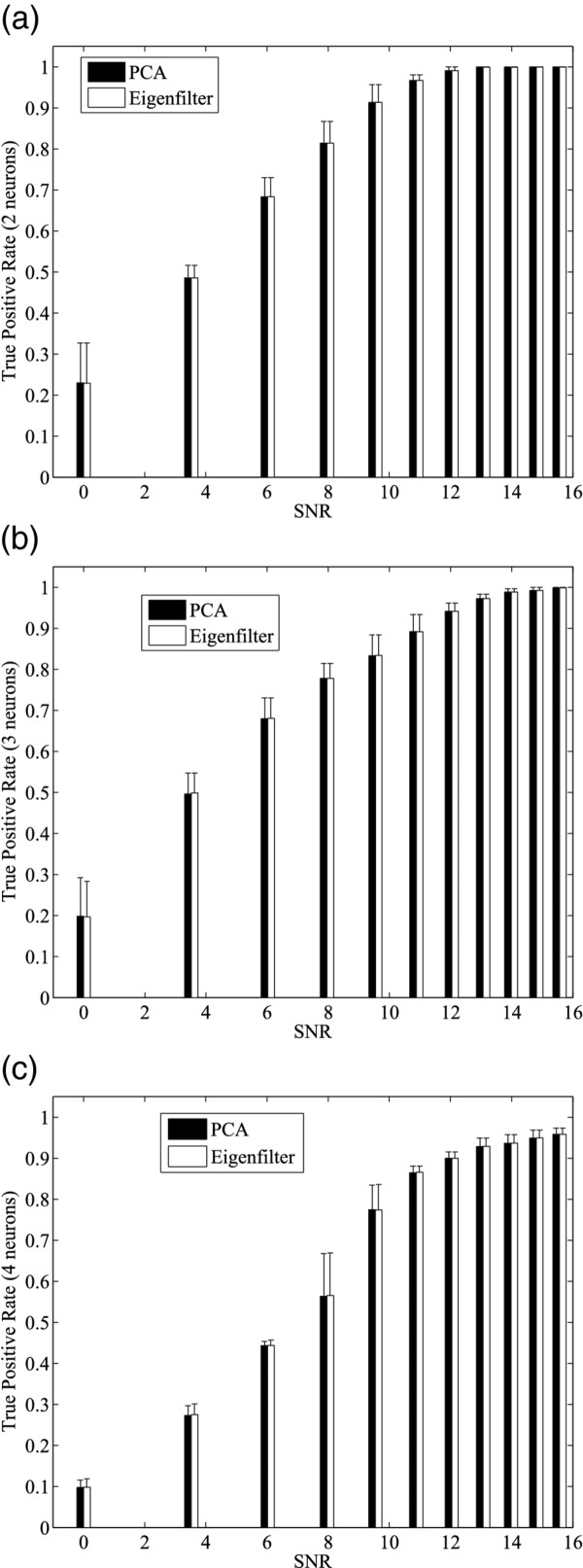
**Evaluation of spike sorting accuracy with varied SNR**. Comparisons between PCA (Matlab princomp) and stream-based Hebbian eigenfilter are based on (a) 2 neruons (b) 3 neruons (c) 4 neurons cases.

**Figure 8:**
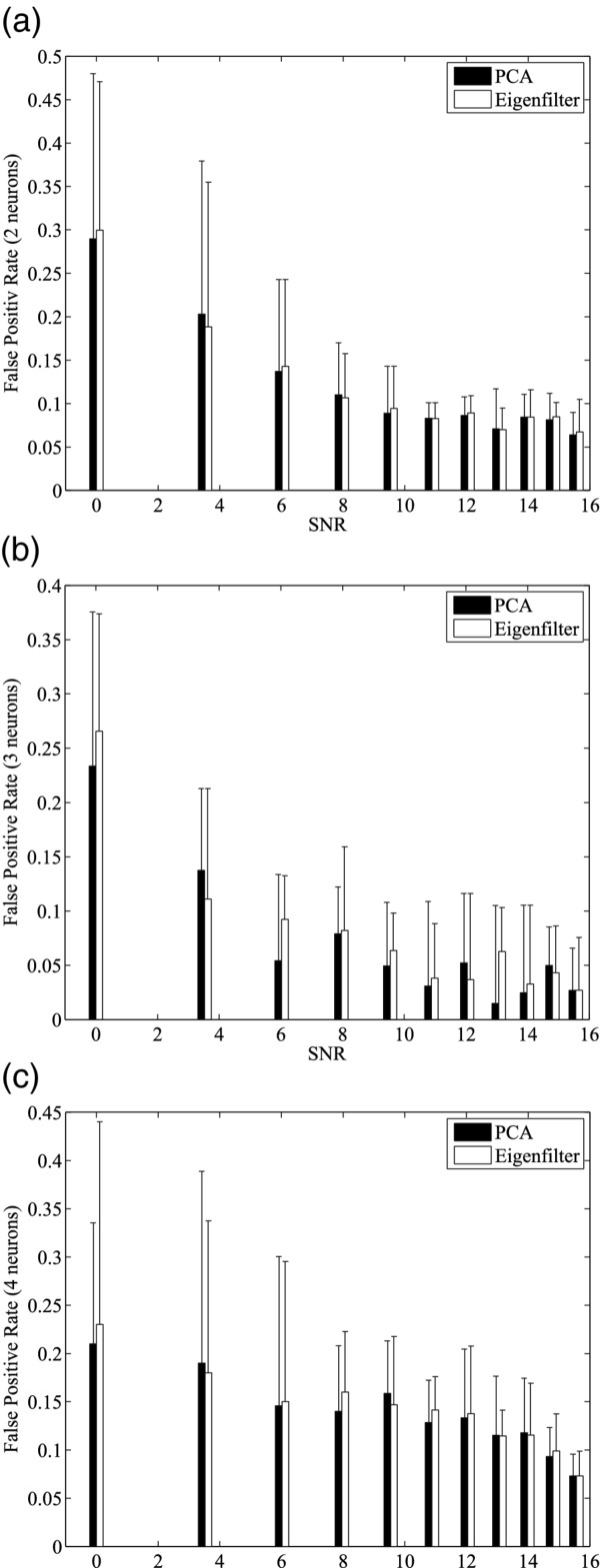
**Evaluation of false positive rate of spike sorting with varied SNR**. Comparisons between stream-based Hebbian eigenfilter and PCA (Matlab princomp) are based on (a) 2 neruons (b) 3 neruons (c) 4 neurons cases.

In addition, the noise immunity of the proposed method was studied. Synthetic spike trains with various noise level and neurons (2 to 4) were generated by synthetic spike tool [29]. Figure 7 shows the relationship between the true positive rate and SNR for spike sorting algorithms using the stream-based Hebbian eigenfilter and Matlab built-in algorithm for PCA, *princomp*. Comparing Figure 7(a), (b) and 7(c), we can see that at the same SNR level, the smaller the neuron number is, the better classification results are. We can also see that there is little difference between stream-based Hebbian eigenfilter and Matlab *princomp *function used for spike sorting. As a result, stream-based eigenfilter has the same effect as other PCA algorithms in spike sorting process. Figure 8 shows the relationship between the false positive rate and SNR for spike sorting algorithms using stream-based Hebbian eigenfilter and Matlab built-in PCA. The false positive rates of spike sorting using the stream-based eigenfilter and Matlab *princomp *function are nearly the same. For both methods, the false positive rate falls as the SNR increases.

### Computational complexity

In this section, the computational complexity of stream-based Hebbian eigenfilter are evaluated and compared with GHA and other conventional PCA algorithms in terms of operation counts and memory consumption. Hebbian and conventional PCA algorithms are all based on matrix computations. The complexity of matrix computations is associated with the dimension of matrixes. Particularly, for spike feature extraction, it is determined by the dimension of aligned neuronal spikes. In our evaluation, we suppose the number of spikes for PCA training is 1024, each spike contains 64 samples, and the first two PCs are calculated.

Conventional PCA algorithms consist of covariance matrix calculation and the eigenvalue decomposition. Three most used eigenvalue decomposition algorithms were evaluated, including orthogonal iteration, QR method and Jacobi method. The three benchmark algorithms are all based on some sort of iterative calculations. We assume that these numerical methods can be converged in 8 iterations in our evaluation. The operation counts of Hebbian eigenfilter and three numerical PCA algorithms are listed in Table 3. The results show that Hebbian eigenfilter reduce 89.8% ~ 95.7% and 86.6% ~ 93.4% addition and multiplication operations, respectively. Besides, Hebbian method does not need division and square root operations, which are complex and costly for hardware implementation.

**Table 3:** Comparison on computational complexity between PCA methods and Hebbian eigenfilter

		**#Add**.	**#Multi**.	**#Div**.	#Square root	#Memory
		**× 10^6^**	**× 10^6^**	**× 10^3^**	**×10^3^**	**(bits) × 10^6^**

Hebbian eigenfilter (HE)		**0.46**	**0.59**	**0**	**0**	**1.05**

Stream-based HE (SHE)		**0.46**	**0.59**	**0**	**0**	**0.0031**

Orthogonal iteration (OI) based PCA		4.51	4.39	0.016	0.016	1.18

QR method based PCA		4.77	4.75	1.1	0.57	1.25

Jacobi method based PCA		10.6	8.94	48.4	322	1.18

Computational	OI vs SHE	89.8%	86.6%	100%	100%	99.7%

complexity improvement	QR vs SHE	90.4%	87.6%	100%	100%	99.8%

(reduction rate)	Jacobi vs SHE	95.7%	93.4%	100%	100%	99.7%

Table 3 also lists the memory consumptions of GHA, stream-based eigenfilter and conventional PCA algorithms. In order to estimate the memory requirement, we suppose that each sample is represented by 16 bits. Because of not requiring covariance matrix and matrixes related to eigenvalue decomposition, GHA needs smaller memory than conventional PCA algorithms. But, large size memory is still needed for mean calculation and the following zero-mean transformation. Through employing stream-based processing that estimates the mean value by using the previous data, stream-based Hebbian algorithm does not require the large memory for buffering neuronal spikes for mean and zero-mean transformation. Thus, it consumes the smallest memory. Compared with GHAand PCA, around 99% memories can be reduced by the stream-based eigenfilter.

### Hardware evaluation

Both non-stream and stream based Hebbian eigenfilter were implemented on Xilinx Spartan6 FPGA (xc6slx75t). Table 4 shows the area and performance of the hardware Hebbian eigenfilter. The learning latency is the time needed to process one spike. Logic consumption is the LUT (look-up-table), which is the basic logic block in FPGA, consumed by the design. Memory consumption is the block RAM (BRAM) consumed by the design, which is the basic memory block in FPGA. The results show that significant memory and power consumption reduction can be achieved by using the streaming approach. Compared with non-streaming eigenfilter, 92% BRAM and 67% power consumption can be saved by streaming method.

**Table 4:** Area and performance comparison between FPGA-based Hebbi an eigenfilter and PCA

	Hebbian eigenfilter	Stream-based HE	PCA	Improvement
	**(HE)**	**(SHE)**	[32]	**(SHE vs PCA)**

Number of slice	897	749	7722	10.3x

Number of BRAM	65	5	65	13x

Power (mW)	7.3	2.4	49.3	20.5x

Learning latency (*μs *)	5.6	5.6	42.4	7.6x

Table 4 also compares the hardware performance between the FPGA-based Hebbian eigenfilter and PCA hardware [32]. Hardware performances are varied if different technologies and devices are employed for the implementations. Especially, hardware performances using ASIC (application specific integrated circuit) and FPGAs are different for a particular design. To obtain a fair comparison, we normalized the performance of the ASIC-based PCA hardware [32] to FPGA equivalent and compared with our implementations. These would provide insightful quantitative evaluations between the different approaches in terms of hardware performance. But these results shouldn't be regarded as specifications of system performances.

Suppose the PCA hardware architectures have the same computing capability as the Hebbian eigenfilter, under the same clock frequency latency will be proportional to the computational complexity. Latency (or delay) results are reported in Table 4. The Hebbian eigenfilter approach has a significant advantage in delay that is 7.6 times faster than the PCA hardware. This is critical to be employed in real-time spike sorting. The Hebbian eigenfilter approach also has significant improvement in hardware resources. Hardware logic resources can be significantly reduced by 10.3 times when compared to the PCA hardware. Finally, we evaluated the power consumption. Results show that the stream-based Hebbian eigenfilter have 20.5 times improvement when compared to the PCA hardware implementations in terms of power consumption.

## Conclusion

This paper presents a novel stream-based Hebbian eigenfilter for spike sorting. It calculates principal components of neuronal spikes based on an effective auto-associative Hebbian learning, which is computationally economical. A stream-based computing scheme is proposed to effectively reduce the memory requirements by utilizing the pseudo-stationarity of neuronal spikes. Evaluation results show that the stream-based eigenfilter is as accurate as conventional PCA methods for spike sorting. The computational complexity of the proposed method is ten times less than that of conventional PCA. By utilizing the streaming method, 92% memory resources and 67% power consumption can be saved when compared with non-streaming eigenfilter. Compared with FPGA-based PCA hardware, the proposed eigenfilter reduces logic resources and power consumption by 10.3 and 20.5 times respectively. The new method enables real-time spike sorting for multi-channels neuro-physiological experiments and can be further utilized by implantable systems for chronic diseases.

## Endnotes

We follow a widely acceptable approach [33,34] to convert the ASIC design into FPGA results. Particularly, FPGA implementation is 4.5 times slower than the corresponding ASIC design. In terms of area, FPGA implementation is 21 times larger than equivalent ASIC design in terms of area, and 128 logic counts in FPGAs equals to 0.82 *mm*^2 ^in 90 *nm *CMOS technology [34]. Also, FPGA consumes 12 times more power than the equivalent ASIC.

## Competing interests

The authors declare that they have no competing interests.

## Authors' contributions

BY, TM and CSP proposed the idea. BY carried out the experiment, implemented the algorithm and hardware and evaluated the algorithm and hardware. BY and TM drafted the manuscript. LS provided the tool for generating synthetic spikes. TM, LS, YHS, XYL and CSP provided critical revision of the manuscript. TM and YHS supervised the study. TM, CSP and XYL obtained the funding. Also, all the author read and approved the final manuscript.
